# Retiring the language of first-line and second-line antiretroviral therapy

**DOI:** 10.1016/S2352-3018(25)00137-7

**Published:** 2025-06-05

**Authors:** Marco Vitoria, Graeme Meintjes, Nathan Ford, Lisa Frigati, Nandita Sugandhi, Alexandra Calmy

**Affiliations:** Global HIV, Hepatitis and STIs Programmes, World Health Organization, Geneva 1211, Switzerland; Institute of Infectious Disease and Molecular Medicine, University of Cape Town, Cape Town, South Africa; Department of Medicine, University of Cape Town, Cape Town, South Africa; Blizard Institute, Faculty of Medicine and Dentistry, Queen Mary University of London, London, UK; Global HIV, Hepatitis and STIs Programmes, World Health Organization, Geneva 1211, Switzerland; Centre for Integrated Data and Epidemiological Research, School of Public Health and Family Medicine, Faculty of Health Sciences, University of Cape Town, Cape Town, South Africa; Department of Paediatrics and Child Health, Stellenbosch University and Tygerberg Hospital, Cape Town, South Africa; Global HIV, Hepatitis and STIs Programmes, World Health Organization, Geneva 1211, Switzerland; HIV Unit, Division of Infectious Diseases, University Hospital Geneva, University of Geneva, Geneva, Switzerland

The first guidelines for providing HIV treatment in resource-limited public health programmes were issued by WHO in 2002. The guidelines recommended seven first line-regimens for adults, with five second-line regimens advised in case of treatment failure, and additional options given on the basis of availability of specific drugs. For children, four first-line regimens were initially recommended, with three second-line regimens suggested if initial treatment was unsuccessful; the options were partly guided by availability of age-appropriate dosing formulations at the time. Updated guidelines in 2006 recognised that second-line therapy was increasingly unsuccessful and that, although third-line and subsequent salvage therapies were a reality in industrialised countries, treatment options in resource-limited settings largely depended on drug availability and cost.

The approach of recommending sequential lines of therapy according to treatment effectiveness began with cancer therapy in the 1970s.^1^ In HIV care, the public health approach, which prioritises a small number of standardised first-line and second-line treatment options, has been transformative in expanding global access to care.^2^ By providing clear guidance to clinicians and programme managers in resource-limited settings—where drug supply chains are fragile and health staff might have less specialised training—this model has strengthened feasibility, effectiveness, equity, and breadth of treatment delivery. As more efficacious, better-tolerated antiretroviral drugs with higher barriers to resistance have become available, the number of treatment options has been progressively rationalised towards combinations that are safe and appropriate to use across the majority of populations, including adults, children, and pregnant women.

Since 2018, WHO guidelines for antiretroviral therapy (ART) recommended dolutegravir-based regimens across different populations.^3^ The fixed-dose combination of tenofovir, lamivudine, and dolutegravir, the current standard of care worldwide for adults and adolescents, is highly effective and very well tolerated; substitutions due to side-effects, virological failure, and emergence of clinically relevant drug resistance are very low. As of December, 2023, an estimated 77% of people with HIV worldwide were receiving ART, with more than 80% of these individuals being treated with tenofovir, lamivudine, and dolutegravir. This treatment was supported by multiple generic licenses and funding support from the US President’s Emergency Plan for AIDS Relief and the Global Fund to Fight AIDS, Tuberculosis and Malaria.^4^

Despite considerable optimisation of antiretroviral drug regimens in recent years, lifelong adherence remains a challenge and, for various reasons, people have interruptions in care.^5^ The resultant gaps in care often make it challenging for care providers to differentiate between individuals presenting to care for the first time and those who are returning to treatment after a period of disengagement. One review estimated that up to 50% of people living with HIV who are thought to be starting treatment are, in fact, reinitiators.^6^ Reconstructing an exact treatment history and tailoring treatment according to previous regimen exposure is challenging from a public health approach, as an increasing number of people have had previous exposure to various antiretroviral regimens. For example, people who might have been receiving treatment for up to 20 years might not recall which regimens they previously received or might not report previous treatment to their care provider for fear of being badly judged for having a history of uninterrupted care; or clinic records might be incomplete or unlinked between different health-care providers, and HIV drug resistance testing to guide individualised treatment decisions might not be feasible.

The global transition to tenofovir, lamivudine, and dolutegravir occurred mostly without regard for previous treatment exposure and, in many countries, without viral load testing when switching patients to the regimen. Although many individuals would have started on tenofovir, lamivudine, and dolutegravir as a first-line regimen, the majority of people now on the treatment have switched to it from a non-nucleoside reverse transcriptase inhibitor or protease inhibitor-based regimen. An unknown proportion of these people were viraemic at the time of this switch (as their viral load was not measured) and, for them, it is effectively a second-line or third-line regimen (if using previous terminology).

Referring to a tenofovir, lamivudine, and dolutegravir regimen with first-line or second-line terminology does little to support clinical decision making or drug procurement. When assessing an individual for HIV treatment, the key information needed consists of four essential aspects: previous exposure to antiretroviral drugs, previous exposure to specific drug classes, history of treatment failure, and treatment tolerability.

The reality in programmatic settings is that the majority of adults and adolescents living with HIV are receiving tenofovir, lamivudine, and dolutegravir, tolerating it well, and maintaining a suppressed HIV viral load. A small proportion of individuals cannot tolerate tenofovir, lamivudine, and dolutegravir, most frequently because of pre-existing renal impairment or tenofovir-related renal toxicity, and need to be on an alternative regimen (eg, abacavir for tenofovir). For people on tenofovir, lamivudine, and dolutegravir who have never been failed by a previous regimen (previously termed first line), the risk of developing dolutegravir resistance is extremely low (≤0·1%)^7^ and tenofovir, lamivudine, and dolutegravir is likely to be highly durable as a regimen. For those who have been failed by a previous regimen (previously termed second line or third line) and are therefore likely to have pre-existing resistance to lamivudine or tenofovir (or both), the risk of developing dolutegravir resistance is greater, but still only 1–2% over a medium-term follow-up period of up to 3 years.^7^

The key questions for guiding appropriate management of patients who have virological failure on tenofovir, lamivudine, and dolutegravir are whether adherence is adequate (most patients with viraemia on tenofovir, lamivudine, and dolutegravir will resuppress with adherence interventions) ^8,9^ and whether they have had (or potentially had) previous treatment failure, which makes the development of dolutegravir resistance possible, though still infrequent. Beyond these two considerations, the use of first-line, second-line, or third-line regimen terminology no longer assists clinical decision making.

We created a classification framework for ART regimens used in programmatic settings to support clinical decision making and to guide strategic planning of ART programmes (figure). First, there are people on tenofovir, lamivudine, and dolutegravir with no previous treatment failure. Second, there are people who switched to TLD after previous treatment failure. For some of these people, virological failure was not documented by an HIV RNA test. Third, there are people who are on an alternative regimen because of tolerance issues. Finally, there is a small group of people who developed resistance to dolutegravir after a virological failure on tenofovir, lamivudine, and dolutegravir, and who have since been switched to protease inhibitor-based therapy. This fourth group is anticipated to increase in size over time.

In the paediatric context, the language of treatment lines has also become outdated, albeit with some important differences compared with adolescents and adults. Children who began treatment within the first month of life will have been exposed to many regimens, including the neonatal regimen of zidovidine, lamivudine, and nevirapine. Before widespread use of dolutegravir, children would have been exposed to a protease inhibitor, a non-nucleoside reverse transcriptase inhibitor, or both. As with adults, children can have periods of ART interruption and might present to new facilities with a new caregiver who is not aware of their treatment history. Abacavir is used in place of tenofovir as a backbone drug for children weighing less than 30 kg, and data have shown that children starting second-line dolutegravir-based ART with an abacavir (or tenofovir) backbone were at lower risk of virological failure than those starting zidovudine.^10^ Therefore, the abacavir, lamivudine, and dolutegravir regimen could be used as a first-line or second-line treatment, making such terminology unhelpful in children.

Given the treatment landscape described, we feel it is time to move away from the terminology of treatment lines. The next update of WHO guidelines will no longer use the first-line, second-line, and or third-line treatment terminology, but will instead recommend initial and subsequent regimens.

## Figures and Tables

**Figure:**
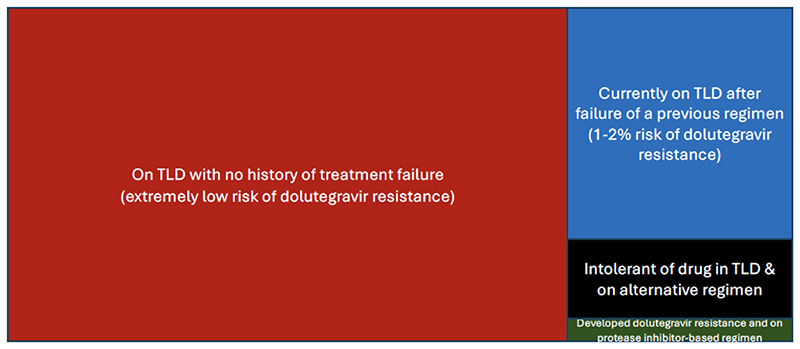
A new categorization of ART regimens (beyond the terms first, second, and third line) in adults and adolescents in programmatic settings Proposed categorisation of the ART regimens people are receiving to aid clinical management decisions and programme planning. Block sizes represent estimated relative differences in prevalence of the four groups that can vary by region; actual block sizes are not reflective of exact, empirically derived proportions. ART=antiretroviral therapy. TLD=tenofovir, lamivudine, and dolutegravir.
